# Current Knowledge of Long Non-Coding RNA HOTAIR in Breast Cancer Progression and Its Application

**DOI:** 10.3390/life11060483

**Published:** 2021-05-26

**Authors:** Yubo Shi, Qingyun Huang, Xinyu Kong, Ruichen Zhao, Xinyue Chen, Yujia Zhai, Lixia Xiong

**Affiliations:** 1 Department of Pathophysiology, Basic Medical College, Nanchang University, Nanchang 330006, China; 15797899116@163.com (Y.S.); 401442718029@email.ncu.edu.cn (Q.H.); xinyu.kong@se17.qmul.ac.uk (X.K.); r.zhao@se17.qmul.ac.uk (R.Z.); 4203119327@email.ncu.edu.cn (X.C.); jp4217118118@qmul.ac.uk (Y.Z.); 2 Queen Mary School, Nanchang University, Nanchang 330006, China; 3 Second Clinical Medical College, Nanchang University, Nanchang 330006, China; 4 Jiangxi Province Key Laboratory of Tumor Pathogenesis and Molecular Pathology, Nanchang 330006, China

**Keywords:** lncRNA, HOTAIR, breast cancer, progression, competitive endogenous RNA

## Abstract

Breast cancer is one of the most devastating cancers with high morbidity and mortality in females worldwide. Breast tumorigenesis and further development present great uncertainty and complexity, and efficient therapeutic approaches still lack. Accumulating evidence indicates HOX transcript antisense intergenic RNA (HOTAIR) is dysregulated in cancers and has emerged as a novel hotspot in the field. In breast cancer, aberrant HOTAIR expression is responsible for advanced tumor progression by regulating multifarious signaling pathways. Besides, HOTAIR may act as competitive endogenous RNA to bind to several microRNAs and suppress their expressions, which can subsequently upregulate the levels of targeted downstream messenger RNAs, thereby leading to further cancer progression. In addition, HOTAIR works as a promising biomarker and predictor for breast cancer patients’ diagnosis or outcome prediction. Recently, HOTAIR is potentially considered to be a drug target. Here, we have summarized the induction of HOTAIR in breast cancer and its impacts on cell proliferation, migration, apoptosis, and therapeutic resistance, as well as elucidating the underlying mechanisms. This review aims to provide new insights into investigations between HOTAIR and breast cancer development and inspire new methods for studying the association in depth.

## 1. Introduction

Globally, breast cancer is estimated to be the most common cancer type (24.5%) occurring in females in accordance with the highest mortality (15.5%) in 2020 [1]. The risk factors of breast cancer include age, family history, estrogen levels, and life style [2]. Additionally, genetic predisposition also largely contributes to breast cancer initiation, including breast cancer gene 1 and 2 (BRCA1 and BRCA2), epidermal growth factor receptor (EGFR), human epidermal growth factor receptor 2 (Her2), phosphatase and tensin homologue deleted on chromosome 10 (PTEN), TP53, c-Myc, and others [2,3]. Breast cancer can be grossly classified into non-invasive breast cancer and invasive breast cancer, but under each unit, more subtypes have been divided. The non-invasive breast cancer contains ductal carcinoma in situ and lobular carcinoma in situ, and the invasive breast cancer is a large group including, typically, invasive lobular carcinoma, invasive ductal carcinoma, and other types [4]. Based on the molecular profiles, it has been more subtly divided into luminal-like type (luminal A and luminal B), basal-like, Her2-positive, and normal type [5,6]. Clinically, breast cancer is also sorted according to the status of estrogen receptors (ERs), progesterone receptors (PRs), and Her2. Given the situation that all the types of receptors are negatively expressed, it is regarded as triple-negative breast cancer (TNBC), which is now a tricky issue to deal with, and patients are always associated with poor prognosis [7].

HOX transcript antisense intergenic RNA (HOTAIR) is a well-studied long non-coding RNA (lncRNA). Elevated HOTAIR expression is constantly observed in many malignancies and is closely related to cancer development [8,9]. In breast cancer cells and tissues, high HOTAIR expression could promote tumor progression and trigger drug resistance. In this review, we summarize the recent advances of HOTAIR in breast cancer, in the hope of providing novel approaches to treating breast cancer.

## 2. Overview of lncRNA and HOTAIR

LncRNAs, together with microRNAs (miRNAs), transfer RNAs (tRNAs), and ribosomal RNAs (rRNAs), are the major entities in the non-coding RNA (ncRNA) family. LncRNAs refer to a group of RNAs with more than 200 nucleotides in length, which are not translated for proteins but remain to be original RNA states; nevertheless, they are functional but largely under investigation. However, recently, it has been recognized that some lncRNAs contain short open reading frames which can encode functional cancer-related peptides [10]. In light of the location relative to encoding genes, they can be categorized as sense lncRNA, antisense lncRNA, intronic lncRNA, bidirectional lncRNA, and long intergenic non-coding RNA [11]. With regard to the action modes, lncRNAs have cis-acting and trans-acting categories. The cis-acting group exerts functions locally near transcriptional loci. By contrast, trans-acting lncRNAs regulate a larger number of genes located on other chromosomes, independent of original sites [12]. LncRNAs are actively involved in chromatin modification and transcriptional and post-transcriptional regulation by means of signal, decoy, guide and scaffold through various interacting homologous RNA, hybridizing DNA, or recruiting proteins [13,14,15]. LncRNAs have highlighting roles in oncology by modulating a variety of signaling pathways and molecules, for instance, p53, nuclear factor kappa B (NF-κB), and phosphoinositide 3-kinase/protein kinase B (PI3K/AKT) [16]. In breast cancer, lncRNAs have dual roles; some can function as tumor suppressors, like MEG3 and ANCR, and others will contribute to cancer progression, such as ARNILA and UCA1 [17,18]. They are involved in cell proliferation, invasion, epidermal-mesenchymal transition (EMT), migration, apoptosis, multiple drug resistance (MDR), and metastasis [19,20]. Collectively, lncRNAs have both suppressive and promotive roles in malignances. 

**Figure 1:**
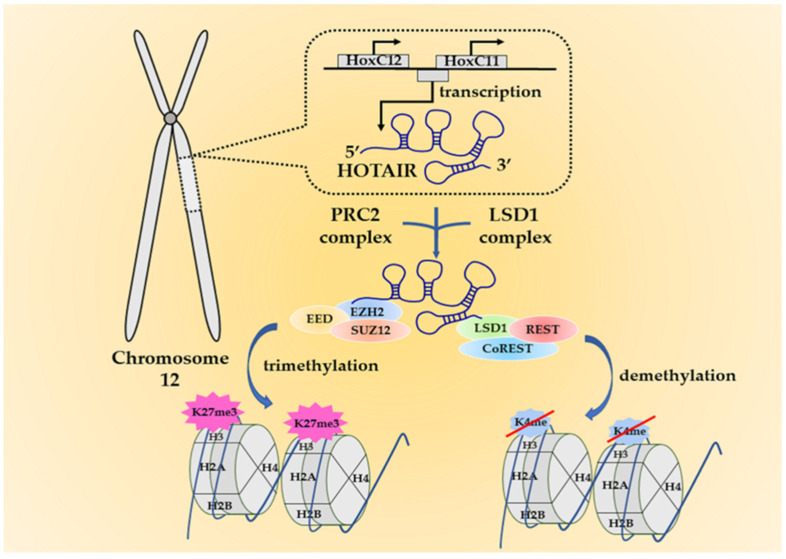
The origin of HOX transcript antisense intergenic RNA (HOTAIR) and its working mechanisms. HOTAIR is an anti-sense lncRNA that is transcribed from the HomeoboxC (HoxC) gene cluster at chromosome 12. It interacts with the polycomb repressive complex 2 (PRC2) complex at the 5′ end and the lysine-specific demethylase 1 (LSD1) complex at the 3′ end to exert the functions. The two complexes can respectively induce H3K27me3 or H3K4 demethylation. These epigenetic alterations eventually lead to gene silencing and may trigger oncogenesis.

In the lncRNA family, HOTAIR is identified as a spliced and polyadenylated transcript with 2158 nucleotides that is transcribed from the anti-sense strand of the HomeoboxC (HoxC) gene locus located on chromosome 12 (Figure 1) [21]. Using a combination of chemical probes, its four-domain experimental structure was depicted several years ago, but until recently, the direct structure of HOTAIR has been unraveled by atomic force microscopy [22,23]. This direct visualization makes it possible for researchers to focus on its mutual interactions with DNA, RNA, or proteins more intensively. HOTAIR is a significant regulator in modulating chromatin status and silencing gene transcriptions. Originally, HOTAIR was found to silence gene expressions on the distinct HoxD cluster by recruiting polycomb repressive complex 2 (PRC2) at the 5′ end [21,24]. PRC2 is a complex with histone methyltransferase activity. It encompasses three core subunits, namely the suppressor of zeste 12 (SUZ12), the embryonic ectoderm development (EED), and the enhancer of zeste homolog 2 (EZH2) [25]. More specifically, there is evidence implying HOTAIR binds PRC2 by directly interacting with the subunit EZH2 [26,27]. EZH2 is the main catalytic factor in the PRC2 complex for histone H3 lysine 27 trimethylation (H3K27me3), a mark usually indicating a status that chromatin is epigenetically repressed [25,28]. HOTAIR depletion restores H3K27 from methylation induced by PRC2 and concomitantly reactivates gene transcription [21,24]. Although in most cases the suppressive role is considered PRC2-dependent, Portoso et al. overexpressed HOTAIR in breast cancer cells, and they detected gene silencing regardless of the PRC2 presence or depletion. It indicates that HOTAIR may not require the PRC2 complex to carry out its repressive roles on gene expression, but the actual mechanism is still uncertain [29]. At the other end—the 3′ domain, HOTAIR binds to the lysine-specific demethylase 1/corepressor of repressor element-1 silencing transcription factor/repressor element-1 silencing transcription factor (LSD1/coREST/REST) complex (LSD1 complex), a demethylase that catalyzes H3K4 demethylation [30]. Hence, HOTAIR has bifunctional regulation on chromatin status by coordinately bridging with the two complexes (Figure 1). HOTAIR is also implicated in post-translational modification by associating with two E3 ubiquitin ligase, namely mex-3 RNA binding family member B (Mex3b) and DAZ interacting zinc finger protein 3 (Dzip3), along with their respective ubiquitination substrates, Ataxin-1 and Snurportin-1. Through the cascade, HOTAIR forms complexes with Mex3b and Dzip3 as a scaffold and degrades the two aforementioned substrates via ubiquitin-mediated proteolysis [31]. 

Gupta et al.’s work lifts understandings of HOTAIR to a new level, which extends the silencing effects genome-wide and demonstrates its roles in cancer progression [32]. Apparently, HOTAIR is a trans-acting lncRNA. HOTAIR overexpression can widely recruit PRC2 to specific gene loci and induce H3K27me3, epigenetically silencing gene expression and driving invasiveness and metastasis along with other malignant potentials. In particular, for breast cancer, BRCA1 associates with EZH2 in a binding region that overlaps with a HOTAIR-binding domain, thus blocking HOTAIR–EZH2 (PRC2) interaction. On the contrary, BRCA1 deregulation, which is universal for breast cancer patients, facilitates the interaction and enhances PRC2-mediated H3K27me3 on specific gene sites, and thus, tumor progression is initiated [33]. HOTAIR is also actively engaged in prostate cancer, glioblastoma, and gynecologic cancers, where it acts as an oncogenic factor [34,35,36]. Besides, HOTAIR is involved in non-cancer pathological processes, including diabetic cardiopathy [37], Parkinson’s disease [38], and rheumatoid arthritis [39]. In the present study, we will focus on HOTAIR’s functions in the progression of breast cancer.

## 3. Enhanced Transcription of HOTAIR in Breast Cancer with Multiple Approaches

Abundant studies have claimed the enrichment of HOTAIR is observed in various cancer cells and tissues [8]. In breast cancer samples, generally, HOTAIR is significantly higher expressed than its adjacent normal tissues [40,41]. It is necessary to figure out how HOTAIR overexpression is induced in breast cancer. Although estrogen is indispensable for mammary development, it turns to a carcinogenic agent under specific situations. Besides, non-estrogen activators are also engaged in the processes. Herein, we highlight the two groups of molecules and attempt to decipher the underlying mechanisms. 

### 3.1. Estrogen/ER Signaling Pathway Is Significant for HOTAIR Induction

**Figure 2:**
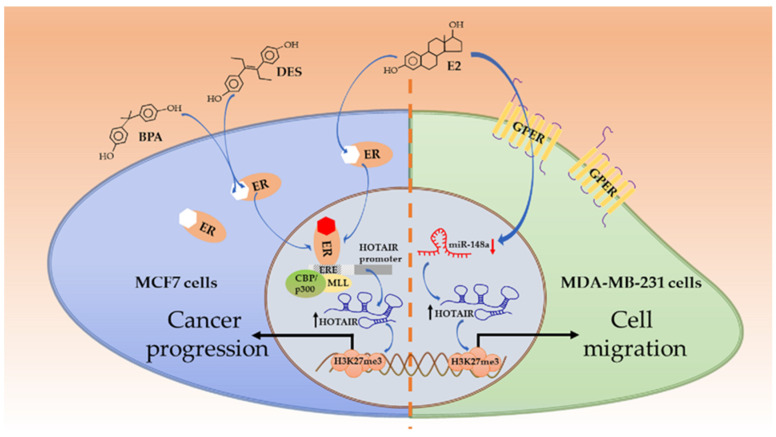
Estrogen/estrogen receptor (ER)-involved HOTAIR induction. E2 and endocrine disruptors (bisphenol-A (BPA); diethylstilbestrol (DES)) resemble in structure and both act on ERs in non-triple-negative breast cancer (TNBC) cells. ERs can bind to estrogen response elements (EREs) on the HOTAIR promoter and enhance its transcription, with the assistance of coactivators MLL and CBP/p300. HOTAIR induces H3K27me3 on targeted genes and propels cancer progression. Alternatively, in TNBC cells, Estradiol (E2) acts on GPERs to reduce miR-148 and increases HOTAIR levels. The HOTAIR-mediated trimethylation of specific genes triggers cell migration for further development.

Estrogen/ER are critically essential for normal development of mammary epithelia and tissues. However, they are also tumorigenic [42]. Estradiol (E2) is a member belonging to the estrogen family. By acting on ER, E2 can activate multiple signaling pathways and control a series of cellular events, for instance, cell proliferation as well as growth of tissues and tumors [43]. HOTAIR is essential for MCF7 cells (ER^+^, PR^+^, and Her2^−^) survival, of which the ablation will lead to growth inhibition and apoptosis acceleration. Being exposed to E2, ERs (both ERα and ERβ) get activated and bind to estrogen response elements (EREs), especially ERE2 and ERE3, in the HOTAIR promoter close to initial transcription sites, and HOTAIR expression is therefore elevated transcriptionally. Some coactivators, such as mixed lineage leukemia (MLL), CREB-binding protein (CBP), and p300, also join in the regulation. However, it is merely observed in ER^+^ cells rather than in ER^−^ ones, such as the TNBC cell line MDA-MB-231 [44]. In TNBC cell lines (such as MDA-MB-231 and BT549), E2 can bypass ER but act on G-protein-coupled estrogen receptor-1 (GPER) to elevate HOTAIR expression, which contributes to cell migration. In this case, E2 does not target the HOTAIR promoter directly, instead, E2 lowers the expression of miR-148a which could bind to HOTAIR via a complementary base pair; thus, the level of HOTAIR is relatively upregulated [45]. As far as we know, whether HOTAIR also targets GPER in non-TNBC cells has not been studied yet. In Xue et al.’s study, however, E2 stimuli have inhibitory roles upon HOTAIR expression in an MCF7 cell line [46]. In fact, when comparing the two independent studies and viewing their results [44,46], it is noticed that HOTAIR up- or downregulation is not linearly related to E2 concentration; in other words, E2 is able to propel HOTAIR transcription at a lower concentration (e.g., 0.01 nM) but repress it at a higher concentration (1 nM). In another independent study, MCF7 cells treated with 10 nM E2 also exhibit decreased HOTAIR expression [47]. These results show that E2 has dual roles in HOTAIR expressions, depending on its concentration. Apart from the oncological capacity of endogenous estrogen, several exogenous xenoestrogens also exhibit similar functions. Some industrial chemical agents (such as bisphenol-A (BPA)) and synthetic estrogenic medication (such as diethylstilbestrol (DES)) are found to be carcinogens, resulting in breast cancer as well as other malignancies [48]. They resemble E2 in a structure (Figure 2) and are known as endocrine-disrupting chemicals. Due to the structural similarity, such agents can mimic E2 and elevate HOTAIR expression in MCF7 cells compared to in the control groups in the same manner through ER-mediated binding to the EREs on the promoter of HOTAIR [49]. These mechanisms have been illustrated in Figure 2.

### 3.2. Non-ER-Mediated Manners Matter in HOTAIR Expression

There are alternative pathways involved in HOTAIR regulation. Jumonji-domain-containing protein 6 (JMJD6) is a phosphatidylserine receptor with arginine demethylase and lysyl hydroxylase activities, and it exerts tumorigenic roles [50]. JMJD6 can strengthen HOTAIR transcription by binding to its promoter in breast cancer cells. However, mutated JMJD6 without lysyl hydroxylase and demethylase activities still interacts with this region but is incapable of altering HOTAIR expression [51]. Her2 is an activator for HOTAIR expression by acting on the effector mitogen-activated protein kinase (MAPK). Her2 and MAPK inhibitors significantly alleviate HOTAIR induction. In turn, the exosomal HOTAIR level reflects the status of Her2 in primary tumoral tissues [52]. In breast cancer cells, the HOTAIR level is decreased following Ras homolog gene family member C (RhoC) knockdown via siRNA transfection, and Rho-associated protein kinase (ROCK) works as the intermediate effector. Essentially, RhoC/ROCK signaling induces the translocation of a transcription cofactor myocardin-related transcription factor A (MRTF-A) into nucleus. MRTF-A forms a complex with a serum response factor (SRF) in the nucleus where they subsequently bind to the CArG box sequence on the HOTAIR promoter, recruit RNA polymerase II and finally proceed HOTAIR transcription [53]. Likewise, the suppression of either kinases Src or p38 could attenuate HOTAIR expression in tumor necrosis factor (TNF)-α-resistant MCF7 cells, leading to decreased cell viability. It was also noticed that HOTAIR has a 69-fold increase in a resistant cell line, which behaves more similarly to basal-like phenotype, in comparison to its parental luminal-like cells. More elevated HOTAIR levels were also discovered in basal-like TNBC cells BT-549 (80-fold) and MDA-MB-157 (60-fold) than in MCF7 cells [54]. These results reveal a regulatory role of HOTAIR in cell type transition, and HOTAIR expressions exhibit tissue specificity.

## 4. HOTAIR Mediates Many Aspects in Regulating Breast Cancer Development

Breast cancer development is a complicated cascade, mainly comprising oncogenesis and enhances cell proliferation and motility and metastasis. Tumor microenvironment, composed of non-cancerous cells, for instance, stromal cells, cancer-associated fibroblast (CAF), and other components, is as important as parenchymal elements to support tumor progression [55]. Substantial evidence suggests HOTAIR is an important regulator in the processes. Hence, we demonstrate HOTAIR functions in the cascade.

### 4.1. HOTAIR and Breast Oncogenesis

As is mentioned above, c-Myc is one of the proto-oncogenes driving tumorigenesis, and HOTAIR is found to participate in this process. HOTAIR itself provides a scaffold to form a complex with hepatitis B X-interacting protein (HBXIP) as well as LSD1, and HBXIP can further interact with c-Myc. All the elements are indispensable and work jointly to drive c-Myc-targeted genes cyclin A, eIF4E, and LDHA expressions at both mRNA and protein levels, which are oncogenic [56]. Overexpression of c-Myc has been suggested to decrease HOTAIR levels in MDA-MB-231 cells, which is depicted in a qPCR-based heatmap [57]. Nevertheless, c-Myc is reported to increase HOTAIR levels by directly binding to its promoter in gallbladder cancer cells, while HOTAIR can elevate c-Myc via the Wnt/β-catenin signaling pathway in leukemia cells [58,59]. Due to the seemingly contradictory results, the association of HOTAIR and c-Myc in malignancies are worthwhile to be further investigated.

### 4.2. HOTAIR Influences Cell Properties in Breast Cancer Stem Cells (BCSCs)

BCSCs play tumor-initiating roles in the malignancies with self-renewal ability. They are typically identified by the high expression of cell surface marker CD44 plus the low expression or absence of CD24 (CD44+/CD24−/low) [60].

HOTAIR is significant for maintaining breast cancer cell stemness and driving other malignant potentials. In HOTAIR-overexpressed cells, HOTAIR is required for EMT and stemness. A broad range of EMT-related genes (ZEB1, SNAIL, and TWIST) and stemness-related genes (SOX1, SOX10, and OCT4) are both activated [61]. In the cancer stem cell (CSC) subpopulation derived from parental MCF7 cells, HOTAIR elevation is detected and responsible for propelling cell proliferation and migration as well as keeping self-renewal capacity. Mechanically, HOTAIR binds to miR-34a and suppresses its expression, and the level of sex-determining region Y-box 2 (SOX2) is thus restored from the inhibited status caused by miR-34a and aids in maintaining stemness [62]. Additionally, HOTAIR targets p53 and interferes with its binding to the promoter of p21, a negative cell cycle regulator. Attenuated p21 expression finally leads to enhanced proliferation in this study [62,63]. It was reported that miR-7 inhibits SET-domain-bifurcated histone lysine methyltransferase 1 (SETDB1), repressing the signal transducer and activator of transcription 3 (STAT3) expression and phosphorylation. STAT3 inactivation reverses EMT in MDA-MB-231 cells and hinders metastasis in BCSC-transfected mice models. However, miR-7 is dramatically suppressed by HOTAIR through HOXD10; thus, the tumor suppressive roles of miR-7 have to be overthrown [64].

### 4.3. HOTAIR Regulates Breast Cancer Cells Proliferation and Apoptosis

Cell proliferation is a cycle containing G1, S, G2, and M phases, usually controlled by a bunch of molecules called cyclins, such as cyclin A, cyclin B, cyclin D, cyclin E, and their partner cyclin-dependent kinases (CDKs), such as CDK1, CDK2, CDK4, and CDK6 [65]. Cell apoptosis is a highly restricted cellular event regulated by a pair of molecules including pro-apoptotic proteins (e.g., Bad and Bax) and anti-apoptotic proteins (e.g., Bcl-2 and Bcl-W) [66]. Cell proliferation and apoptosis are under dynamic equilibrium, whereas strengthened proliferation and prolonged survival will further enable tumor growth.

HOTAIR can disrupt the relative balance by controlling multiple pathways. The protein high mobility group AT-hook 2 (HMGA2) is oncogenic. Elevated HOTAIR levels in breast cancer cells can facilitate HMGA2 expression by targeting and arresting miR-20a-5p levels, which ultimately contributes to cell proliferation and survival. The disruption of the HOTAIR/miR-20a-5p/HMGA2 axis alleviates tumor growth and shrinks tumor volume in vivo [67]. Bcl-w is a member of the Bcl-2 family that promotes cell survival. HOTAIR can elevate the expression of Bcl-w in breast cancer cells MCF7 and T47D, which show stronger proliferation afterwards. During this process, miR-206 serves as an intermediate that can be repressed by HOTAIR via complementary base pairing but reduces Bcl-w levels via binding to the 3′ untranslated region (3′ UTR) of Bcl-w mRNA [68]. HOTAIR also sequesters miR-601 and represses it, consequently leading to the induction of zinc finger E-box-binding homeobox 1 (ZEB1), a pro-oncogenic protein that impels cell proliferation and migration in MCF7 and MDA-MB-231 cells. HOTAIR knockdown hinders tumor growth in vivo [69]. HOTAIR is also regulated by other ncRNAs to regulate breast cancer cell proliferation. It is noticed that HOTAIR is upregulated in breast cancer tissues, while another lncRNA, downregulated in hepatocellular carcinoma (DRHC), is actually downregulated. In TNBC BT549 and HCC70 cells, DRHC is identified as an upstream inhibitor of HOTAIR that reduces cell proliferation by lowering HOTAIR expression [70]. HOTAIR ablation can significantly upregulate p53 but downregulate AKT, c-Jun N-terminal kinase (JNK) and matrix metalloprotease (MMP)-2/9; thereafter, they inhibit proliferation while promoting the apoptosis of MCF7 cells. The aberrant p53/AKT/JNK axis also suppresses their migratory and invasive abilities [71]. 

### 4.4. HOTAIR Regulates Cell Migration and Metastasis

Breast cancer patients mainly die from tumor metastasis, the most common locations of which are bone, lung, brain, and liver [72]. Metastasis is grossly a consequence of the process that the primary tumor spreads and forms a second lesion in distance. The metastatic cascade consists of a series of cellular events, including angiogenesis, EMT, and cell invasion and migration [73]. EMT is one of the key steps, during which original epithelial cells are transformed into mesenchymal-like cells, enabling them to invade and migrate [74]. It is regulated by complicated signaling pathways that have not been entirely elucidated so far. 

HOTAIR is a critical molecule in cell invasion and migration. In SKBR3 and MCF7 cells, HOTAIR binds to and alleviates miR-129-5p, inducing invasion, migration, and EMT by driving frizzled-7 (FZD7) expression [41]. On the contrary, HOTAIR expression can be positively regulated by the ectopic overexpression of upstream miR-146a-5p, and cell lines BT549 and HCC70 are thus conferred with enforced migration and invasion capacity [75]. In the process of migration, one of the HOTAIR-targeted gene is carbohydrate sulfotransferase 15 (CHST15), which encodes the protein catalyzing the biosynthesis of chondroitin sulfate-E, a member belonging to glycosaminoglycan expressed on cell surface. HOTIAR can promote CHST15 expression and enhance invasiveness in vitro and lung metastasis in vivo. Besides, HOTAIR-induced CHST15 transcription also helps maintain the stemness of breast cancer cells [76]. Vasculogenic mimicry (VM) is an approach adapted by tumor cells to develop vessel-like channels independent of endothelial cells for nutrients [77]. A recent study shows HOTAIR expression is upregulated under hypoxia conditions, which also gives rise to VM. Contrarily, HOTAIR deficiency attenuates cell migration and VM by upregulating miR-204. MiR-204 is an intermediate that is sponged and negatively regulated by HOTAIR but inhibits migration-related protein focal adhesion kinase (FAK) and plays suppressive roles [78]. Apart from breast cancer cells, HOTAIR can also be induced by CAF, a pivotal component in the tumor microenvironment. It was reported that CAF-secreted TGF-β1 can effectively stimulate HOTAIR expression in breast cancer cells by activating the TGF-β/Smad pathway. HOTAIR subsequently activates CDK5 by inducing H3K27me3 on the promoter of CDK5 regulatory subunit-associated protein 1 (CDK5RAP1), a repressor of CDK5, and epigenetically silences the gene. CDK5 elevation finally leads to EMT in vitro and distant metastasis in vivo [79,80]. Meredith et al. identified and separated a binding protein called heterogeneous nuclear ribonucleoprotein (hnRNP) A2/B1, using RNA pulldown assays and quantitative mass spectrometry. A2/B1 (B1 preferentially) assists matchmaking between HOTAIR and targeted transcripts by bridging them altogether. The A2/B1–HOTAIR interaction is important for maintaining HOTAIR-induced H3K27me3 of targeted genes and invasiveness in MDA-MB-231 cells [81]. In a cohort of 163 TNBC clinical samples, high tumoral HOTAIR is significantly associated with lymph node (LN) metastasis. Intriguingly, it is also demonstrated that HOTAIR levels are strongly correlated to androgen receptor (AR) status in breast cancer [82]. Previous reports showed that AR is expressed in approximately 60.5% of breast cancer (4658/7693) and is involved in multiple signaling pathways to modulate cellular processes; thus, it is a promising drug target in clinical applications [83,84].

## 5. HOTAIR Participates in Breast Cancer Management

**Figure 3:**
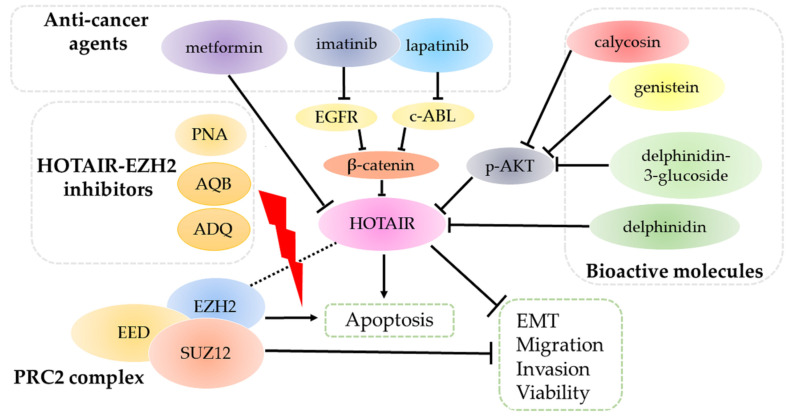
HOTAIR is the target of several sets of molecules. Anti-cancer agents (metformin, imatinib, and lapatinib) and bioactive molecules (calycosin, genistein, delphinidin, and delphinidin-3-glucoside) target and inhibit HOTAIR through multiple signal pathways. Consequently, HOTAIR ablation suppresses tumor progression by reversing epidermal-mesenchymal transition (EMT) and decreasing migration, invasion and viability, along with promoting apoptosis. HOTAIR-EZH2 inhibitors PNA, AQB, and ADQ can disrupt the interactions, thus blocking the binding of the PRC2 complex to HOTAIR, which eventually attenuate cancer development.

The treatments of breast cancer have evolved for decades, including surgeries, chemotherapies, radiotherapies, immunotherapies, endocrine therapies, and targeted therapies [85,86,87]. The exact treatment approach is determined according to tumor size, stages, drug responses, and other valuable pathological parameters. As HOTAIR is constantly overexpressed in breast cancer and plays broad roles in leading to deterioration, it is thus proposed as an ideal drug target. As illustrated in Figure 3, three sets of molecules are reported to attenuate tumor progression through HOTAIR.

### 5.1. Chemotherapeutic Agents

Imatinib and lapatinib are targeted drugs, targeting tyrosine kinases c-Abl and EGFR, respectively [88]. In cellular levels, the combination of the two drugs significantly suppresses HOTAIR expression by inhibiting nuclear β-catenin and blocking its recruitment to the HOTAIR promoter. However, ectopic HOTAIR introduction will enhance resistance against co-treatment [89]. The anti-diabetic medication metformin has also been conferred with anti-cancer roles [90]. Recently, metformin was reported to diminish cell viability by targeting HOTAIR in breast cancer cells. Golshan et al. demonstrated that metformin treatment induces dose-dependent DNA methylation on the HOTAIR promoter and leads to its decrease, as well as suppressing cell motility and reversing EMT in MDA-MB-231 cells [91].

### 5.2. HOTAIR–EZH2 Inhibitors

To carry out its functions, HOTAIR scaffolds the PRC2 complex by directly linking to the EZH2 subunit. A new peptide nucleic acid (PNA)-based therapy has been recently proposed to disrupt this conjugation in breast and ovarian cancer cells. PNA binds HOTAIR single-stranded region, which leads to inhibited clonogenic survival and increased chemosensitivity to cisplatin, at least by inhibiting NF-κB activation and expressions of MMP-9 and interleukin-6. PNA only abrogates HOTAIR–EZH2 interaction but does not influence HOTAIR levels significantly [92]. A research group selected two small compounds, namely AC1NOD4Q (ADQ) and AC1Q3QWB (AQB) by high-throughput sequencing that interfere with a HOTAIR–EZH2 complex in MDA-MB-231 cells [93,94]. Such molecules work in a similar manner that interact with HOTAIR to block its association with EZH2 and prevent PRC2 recruitment. Thus, they can suppress β-catenin levels by increasing nemo-like kinase (NLK) and adenomatous polyposis coli 2 (APC2) expressions, respectively, which eventually prohibit tumor progression in vivo. In the process, HOTAIR is not altered, but H3K27me3 is significantly decreased following either treatment, which suggests the restart of targeted gene transcriptions.

### 5.3. Natural Bioactive Molecules

Natural products have critical tumor suppressive roles [95]. Calycosin and genistein are both isoflavone family members showing inhibitory roles against various cancers. In MCF7 cells, they are capable of suppressing AKT activation, thus decreasing downstream HOTAIR expression. These alterations are responsible for inhibited cell proliferation and promoted apoptosis [96]. Delphinidin is one of anthocyanidins stored in fruits and vegetables with anti-cancer effects. Delphinidin administration can decrease HOTAIR expression in MDA-MB-231/453 and MCF7 cells, of which the suppression subsequently upregulates miR-34a. MiR-34a can inactivate the Wnt/β-catenin pathway to inhibit cell proliferation as well as invasion, and collectively, breast carcinogenesis in vivo [97]. Its derivative, delphinidin-3-glucoside, is also valid in decreasing HOTAIR levels to inhibit breast carcinogenesis through the AKT/ interferon regulatory factor (IRF)-1 signaling axis [98]. 

## 6. HOTAIR Induces Resistance against Chemo-Radiotherapies

The occurrence of chemoresistance and radioresistance can be ascribed as follows: increased drug efflux due to altered membrane status, enzyme-mediated drug inactivation, aberrant receptor quantity and binding ability, disruption of dynamic equilibrium between proliferation and apoptosis, strengthened DNA repair, CSC, and tumor environment. They are controlled by many different signaling pathways. LncRNAs are recognized to induce therapeutic resistance in breast cancer by mainly altering gene expressions that control such processes [99,100,101,102,103]. 

### 6.1. HOTAIR and Chemoresistance

The presence of HOTAIR is closely associated with chemoresistance. By treating MCF7 cells with long-term tamoxifen, the cell line exhibits drug resistance against it, during which HOTAIR is remarkably elevated. As a consequence, cell growth is stimulated [46]. Some studies confirmed that HOTAIR depletion would contribute to the suppression of drug resistance. In doxorubicin-resistant MCF7 cells, levels of pro-apoptotic proteins caspase-3 and Bax are increased following HOTAIR knockdown, while anti-apoptotic protein Bcl-2 is reduced. HOTAIR ablation also downregulates MDR-related proteins, such as multidrug-resistance-associated protein 1 (MRP1) and multidrug resistance protein 1 (MDR1). They may cooperate to slow down proliferative rates and accelerate cell death. Meanwhile, HOTAIR silencing reduces phosphorylated expressions of PI3K, AKT, and mammalian target of rapamycin (mTOR), suggesting the PI3K/AKT/mTOR signaling pathway may play a role in HOTAIR-induced doxorubicin resistance [104]. The trastuzumab-resistant SK-BR-3 cell line expresses higher HOTAIR compared with the sensitive group. Owing to HOTAIR knockdown, the resistant cells respond to trastuzumab and loss viability. Concordantly, PTEN is upregulated due to demethylation, and TGF-β is downregulated due to methylation. They subsequently deactivate downstream genes (such as Snail and Vimentin) to suppress invasion. HOTAIR depletion also represses cyclin D1 in resistant cells, which arrests cells in G0/G1 phase and alleviates proliferation [105].

### 6.2. HOTAIR and Radioresistance

Radioresistance is another obstacle confronted in clinics. HOTAIR is an essential mediator in the process. After transfection, MDA-MB-231 cells show high HOTAIR expression. As a result, it leads to radioresistance indicated by active proliferation after irradiation, which probably results from targeting and downregulating HOXD 10 as well as increasing phosphorylated AKT but decreasing phosphorylated Bad [106]. Whether HOXD10 can regulate AKT/Bad pathways is uncertain based on the existing data from this report. However, HOXD10 was previously demonstrated to be an upstream negative regulator of the PI3K/AKT pathway in cholangiocellular carcinoma progression, which may also apply to breast cancer, but it needs to be further confirmed [107]. Alternatively, HOTAIR induces heat shock protein family A member 1A (HSPA1A) expression, a stress-associated protein, which endows radioresistance in breast cancer. HSPA1A could be restrained by miR-449b-5p, which potentially binds to the 3′UTR of HSPA1A mRNA, and is thus downregulated. Nevertheless, miR-449b-5p acts as a sponge that is sequestered by HOTAIR through base-pairing reaction and restores HSPA1A expression [108]. In the most recent study, increased DNA repair regulators, such as DNA-dependent protein kinase (DNA-DK), ataxia-telangiectasia mutated (ATM), and Ku70/80, as well as radioresistance, are observed in a subset of HOTAIR-overexpressed MCF7 cells, indicating an intrinsic DNA repair action induced by HOTAIR. It can be taken as a mechanism to explain how HOTAIR triggers radioresistance. This result can be converted by EZH2 inhibitors UNC1999 and EPZ005687, suggesting a role of EZH2 in this process, which is later discovered to be recruited by HOTAIR and driven to the promoter of Myc gene [109]. Reversely, HOTAIR knockdown leads to the upregulation of miR-218 in MCF-7, SKBR3, and MDA-MB-231 cell lines. Thus, the ionizing radiation causes more DNA damages, slowing down proliferative rates by arresting cells in G2/M and inducing intensive apoptosis by activating pro-apoptotic molecules Bax and caspase-3 [40]. To our knowledge, no clinical trials upon HOTAIR expression and radiotherapy effects have been available so far. This result at least indicates that patients with high HOTAIR are not suggested to have radiotherapy solely but a mixture of other approaches is demanded.

## 7. HOTAIR Is a Good Candidate of Diagnostic and Prognostic Biomarker

In oncology, biomarkers refer to a group of measurable factors including proteins and lncRNAs that are essential for risk assessment, screening, differential diagnosis, prognosis, and response prediction [110]. Novel biomarkers have been continuously detected and discovered, in order to meet high demands of sensitivity and specificity. Increased HOTAIR levels can either be detected from tumoral tissues or serum samples, and its roles of being biomarker for the diagnosis and prognosis of breast cancer have been gradually recognized (Table 1).

### 7.1. Diagnosis

Some studies reveal HOTAIR to be a diagnostic biomarker in breast cancer. Generally, HOTAIR expression is significantly elevated in patients’ serum than in healthy ones, which can be regarded as a potential diagnostic biomarker of breast cancer [111]. In a clinical trial, results indicated that HOTAIR expression was significantly higher in cancerous tissues and patients’ serum samples than in adjacent non-cancerous tissues and healthy individuals, respectively. Especially in plasma, high HOTAIR levels were associated with LN metastasis (*p* = 0.018) and Her2 presence (*p* = 0.006), which is consistent with a previous one. In comparison to another two commonly used diagnostic molecules carcinoembryonic antigen (CEA) and cancer antigen (CA) 15-3, HOTAIR had a stronger capacity for breast cancer diagnosis with an area under curve (AUC) of 0.80, a 69.2% sensitivity, and a 93.3% specificity. Moreover, HOTAIR levels were remarkably reduced after surgery which confers a role of HOTAIR in monitoring tumor status delicately (*p* = 0.029) [112]. The diagnostic and monitoring values were further strengthened in an independent study using serum exosomal HOTAIR as the sample [113]. Unexpectedly, Zhang et al. reported circulating HOTAIR DNA is shown to be the main form in patient sera that significantly distinguishes patients from healthy ones (*p* < 0.01) [114]. In that study, DNase I was applied to digesting the DNA sequence in serum samples, where HOTAIR was undetectable following the treatment. In contrast, this step was skipped in the other two independent studies. It is probable that they detected the mixture of HOTAIR DNA and RNA transcripts together.

### 7.2. Prognosis

Exosomes are small-sized extracellular vesicles that can be secreted by many cancer cells. Proteins and nucleic acids are contained inside vesicles, which can indicate the status of the cells and work as biomarkers and therapeutic targets [119]. In 20 patients’ serums, exosomal HOTAIR that was derived from primary tumor lesions was significantly higher expressed than in the healthy group (*p* < 0.001). Additionally, patients with high exosomal HOTAIR were less responsive to neoadjuvant chemotherapy (taxane+anthracycline+cyclophosphamide) and endocrine therapy (tamoxifen). Consequently, this may explain why higher exosomal HOTAIR may associate with poorer disease-free survival (DFS; *p* = 0.048) and overall survival (OS; *p* = 0.046) than the lowly expressed controls [113]. Likewise, one trial evaluated the association of circulating HOTAIR with drug efficacy (anthracycline and taxane) and survival in 112 breast cancer patients’ samples, and elevated HOTAIR levels were found to predict alleviated drug response (*p* = 0.017) and shorter DFS (*p* = 0.012) [115]. Recent studies claimed the association between HOTAIR level and patient’s survival is ER-status-specific. Patients with metastasis were more likely to have higher HOTAIR expression, and they had a poorer metastasis-free survival (MFS) rate (*p* = 0.012) than the low-expression group. Especially in the ER-positive group, high HOTAIR was more significantly associated with worse MFS (*p* = 0.0086), whereas it was insignificant in the ER-negative group [116]. By contrast, Gökmen-Polar et al. drew an opposed conclusion. In their research, higher HOTAIR expression solely indicated shorter OS in ER-negative breast cancer (*p* = 0.018) or in the ER-negative plus LN metastasis-positive (*p* = 0.021) group. In general, however, there is no such relationship in ER-positive samples, regardless of LN metastasis presence or absence [117]. The distinct conclusions may be owing to the two prognosis values, MFS and OS. Besides, Gökmen-Polar’s study recruited both LN-positive plus -negative cases, whereas the other one excluded the LN-positive group. Conversely, by multivariate analyses, higher HOTAIR patients were found to have lower potentials of relapse (*p* = 0.016) or death (*p* = 0.022) than the lowly expressed ones, while it was not observed using univariate analyses [115]. On the contrary, negative results have also been proposed. Another study evaluated the association of HOTAIR with DFS and OS, which is insignificant [82]. The reason might be that they administrated only TNBC patients in the trial, while other trials contained more molecular subtypes. In addition, the size of case volume may develop a bias.

## 8. HOTAIR Single Nucleotide Polymorphisms (SNPs) and the Susceptibility of Breast Cancer

SNP is the most abundant genetic variation (larger than 1%) in population [120]. SNPs influence the risk of developing diseases or altering pathological parameters, which may vary among human races. Several SNPs of HOTAIR, including rs12826786, rs920778, rs1899663, and rs4759314 (Table 2), have determined that they are linked to breast cancer susceptibility and other clinical values [121,122,123,124,125].

For rs12826786, individuals carrying T allele have more elevated risk of developing breast cancer than C allele carriers [121,124]. It has been demonstrated that rs4759314 with the replacement of A to G is not linked to breast cancer susceptibility in Iranian [124]. However, a protective effect was observed in Chinese, although limited to a subgroup (menopause age: ≤50) [125]. Because of human races diversity, rs1899663 T allele in Indian is a dangerous factor, while it is a protective factor in Iranian and Chinese [123,124,125]. Similarly, rs920778 T>C is associated with higher risk of breast cancer in Turkish, Indian and Iranian, but Yan et al. demonstrated T allele is a risk factor in Chinese [122,123,124,125]. Especially in Turkish, Bayram et al. revealed rs12826786 and rs920778 are associated with worse clinicopathological parameters, including larger tumor size, advanced stage, poor grade, and distant metastasis [121,122]. Further studies are still required, such as investigating in a larger scale and containing more human races and case numbers. These results will greatly strengthen and promote breast cancer therapies based on precision medicine.

## 9. Discussion and Conclusion Remarks

HOTAIR is an important scaffold to regulate chromatin states and silence gene transcriptions through epigenetic modifications. It has been found tightly correlated to breast cancer development by enhancing proliferation, promoting migration, attenuating apoptosis and triggering resistance against therapies. The depletion of HOTAIR can reverse these oncogenic effects and block further tumor progression. In clinical patients’ samples, elevated HOTAIR is significantly associated with LN metastasis and distant metastasis in human body, which indicates a poorer prognosis. Besides, some studies indicate several SNPs in HOTAIR can influence breast cancer risk. Due to its multifaceted roles, it is thus suggested to be a promising drug target. In vitro studies have shown that anti-cancer agents (lapatinib, imatinib, and metformin), bioactive molecules (calycosin, genistein, delphinidin, and delphinidin-3-glucoside), and HOTAIR-EZH2 inhibitors (PNA, ADQ, and AQB) can target HOTAIR and exert anti-tumor activities by lowering its expression or disassociating it from the PRC2 complex.

Among substantial HOTAIR-engaged underlying mechanisms, a larger proportion is achieved by binding to other miRNAs and regulating substrate mRNAs. Mechanically, lncRNAs and mRNAs competitively bind to one single type of miRNA by microRNA response elements (MREs). This is currently regarded as the competitive endogenous RNA (ceRNA) theory [126]. HOTAIR sponges miRNAs to sequester mRNAs and inhibits their transcriptions. We have summarized HOTAIR–miRNA–mRNA axes in breast cancer progression (Table 3). They widely participate in breast cancer cell stemness, proliferation, migration, invasion, apoptosis, and radioresistance. Accordingly, targeting these networks may provide novel insights for breast cancer treatments. As HOTAIR interacts with miRNAs via complementary base pairing, they form reciprocal feedback loops to repressively regulate the levels of each other. However, ectopic miR-146a-5p and lncRNA DRHC can up- and downregulate HOTAIR expressions, respectively, while HOTAIR overexpression fails to significantly influence their levels. Previously, HOTAIR and miR-130a were reported to bind to the same RNA-induced silencing complex (RISC) and thus formed the reciprocal loop in gallbladder cancer cells [58]. Based on this reason, it is assumed that the unidirectional regulation is attributed to the RISC absence. Additionally, miRNAs and lncRNAs varieties and cell specificity cannot be excluded.

From a therapeutic perspective, HOTAIR expression can be constrained by several molecules, and cells exhibit reduced malignant potentials (Figure 3). Therefore, HOTAIR can be a promising drug target theoretically, even though specific drugs have not been developed as yet. Concomitantly, chemoresistance takes place as drug dose and time increase. HOTAIR is an inducer of chemoresistance by regulating a variety of signaling pathways. Hence, how to develop a HOTAIR-targeted drug without triggering chemoresistance in breast cancer remains a problem. HOTAIR also induces radioresistance in multiple breast cell lines, the depletion of which can resensitize such cells to irradiation. Nevertheless, direct clinical data on the association between HOTAIR and radiotherapy responses still lack, which requires more explorations.

Liquid biopsy has emerged as a novel and non-invasive tool in cancer diagnosis [127]. Some studies highlight that increased plasma HOTAIR expressions are in favor of breast cancer diagnosis. Notably, one of them demonstrates that plasma HOTAIR had higher ability (AUC: 0.80; sensitivity: 69.2%; specificity: 93.3%) than CEA (AUC: 0.50; sensitivity: 65.4%; specificity: 50.0%) and CE15-3 (AUC: 0.65; sensitivity: 73.1%; specificity: 60.0%) in the diagnosis [112]. Accordingly, a larger volume of testing samples should be recruited so as to verify and solidify the findings. In one evaluation, it is surprising to find that HOTAIR DNA is an actual fragment exerting diagnostic values, rather than its RNA transcript [114]. Furthermore, both serum and tumoral HOTAIR were found to predict patients’ survival as well as other pathological parameters, even though they show tissue specificity (Table 1). However, the results are challenged by Collina et al., who stated HOTAIR expression and survival were insignificantly associated in a group of 163 TNBC patients [82]. Owing to the complexity of breast cancer, the uncertainty of HOTAIR form, and the discrepancy of the statistic method, the potential clinical significance has still not been integrated.

Although large work has been conducted to elucidate the relationship between HOTAIR and breast cancer progression at present, the recognition upon HOTAIR itself and HOTAIR regulatory networks remains poor. Further investigations are still demanded in order to raise new strategies for breast cancer management on the basis of HOTAIR.

## Figures and Tables

**Table 1:** Tumoral or plasma HOTAIR works as diagnostic or prognostic biomarkers.

Sample	Subtype	Significant Values	Reference
plasma HOTAIR	/	diagnosis	[111]
plasma HOTAIR	79 infiltrating ductal; 7 ductal; 2 mucinous	LN metastasis; ER/Her2/ER+PR+Her2-positive; diagnosis	[112]
exosomal HOTAIR	11 invasive ductal; 4 others; 5 invasive lobular	diagnosis; poor prognosis	[113]
plasma HOTAIR DNA	85 invasive ductal; 2 invasive lobular; 2 mixed invasive; 11 others	diagnosis	[114]
plasma HOTAIR	112 invasive	tumor size/grade/relapse; lymph node (LN) metastasis; poor clinical response/prognosis	[115]
tumoral HOTAIR	139 ductal; 24 non-ductal	LN metastasis; AR-positive	[82]
tumoral HOTAIR	131 invasive ductal; 4 mucinous; 18 invasive lobular; 11 others	poor prognosis (in total and ER-positive)	[116]
tumoral HOTAIR	133 invasive	poor prognosis (in ER-negative)	[117]
tumoral HOTAIR	219 ductal; 56 lobular; 35 mixed; 38 others	lower relapse or death	[118]

**Table 2:** Common single nucleotide polymorphisms (SNPs) that influence breast cancer susceptibility and progression.

Item	Nationalities	SNP	Genotype	Clinical Features	Reference
Bayram et al.	Turkish	rs12826786 C>T	TT	increased breast cancer risk/larger tumor size/advanced stage/poor histological grade/distant metastasis	[121]
Bayram et al.	Turkish	rs920778 T>C	CC	increased breast cancer risk/larger tumor size/advanced stage/poor histological grade/distant metastasis	[122]
Rajagopal et al.	Indian	rs1899663 G>T	GT + TT	increased breast cancer risk	[123]
		rs920778 T>C	TC/TC + CC	increased breast cancer risk	
Hassanzarei et al.	Iranian	rs920778 T>C	TC/CC/TC + CC	increased breast cancer risk	[124]
		rs12826786 T>C	TC/CC/TC + CC	decreased breast cancer risk	
		rs4759314 A>G	AG	Not associated	
		rs1899663 G>T	GT/GT + TT	decreased breast cancer risk	
Yan et al.	Chinese	rs1899663 G>T	GT + TT	decreased breast cancer risk (menarche > 14 or pregnancies > 2)	[125]
		rs4759314 A>G	AG + GG	decreased breast cancer risk (menopause ≤ 50)	
		rs920778 C>T	TC + TT	increased breast cancer risk (abortion > 2)	

**Table 3:** HOTAIR–miRNA–mRNA axes in breast cancer progression.

LncRNA	MiRNA	mRNA	Cell Lines	Roles	Reference
HOTAIR ↑	miR-34a ↓	SOX2 ↑	MCF7-derived BCSC	cancer stemness ↑	[62]
HOTAIR ↓	miR-7 ↑	SETDB1 ↓	MDA-MB-231, MCF7 and their BCSC	invasion, migration, metastasis ↓	[64]
HOTAIR ↑	miR-20a-5p ↓	HMGA2 ↑	MDA-MB-231	proliferation, migration ↑; apoptosis ↓	[67]
HOTAIR ↑	miR-206 ↓	Bcl-w ↑	MCF7, T47D	proliferation ↑	[68]
HOTAIR ↑	miR-601 ↓	ZEB1 ↑	MCF7, MDA-MB-231	invasion, migration ↑	[69]
HOTAIR ↑	miR-129-5p ↓	FZD7 ↑	SKBR3, MCF7	invasion, migration ↑	[41]
HOTAIR ↓	miR-204 ↑	FAK ↓	MDA-MB-231, Hs-578T	migration, vasculogenic mimicry ↓	[78]
HOTAIR ↓	miR-34a ↑	β-catenin ↓	MDA-MB-231/453, MCF7	proliferation and mobility ↓	[97]
HOTAIR ↑	miR-449b-5p ↓	HSPA1A ↑	MDA-MB-231, MCF7	radioresistance ↑	[108]
HOTAIR ↓	miR-218 ↑	/	MCF7, SKBR3, MDA-MB-231	radiosensitivity ↑	[40]

## Data Availability

Not applicable.
